# Ultrasound-Guided Sciatic and Saphenous Nerve Blocks Enhance Perioperative Analgesia in Sheep Undergoing Experimental Orthopaedic Hindlimb Surgery

**DOI:** 10.3390/vetsci13040318

**Published:** 2026-03-26

**Authors:** Oliver Rodriguez, Pedro Figueirinhas, Daniela Vazquez, Sara Del-Rosario, Yeray Brito-Casillas, Sergio Martin, Andrea Paolini, Anabel Mateo-Cebrián, Raquel Rodríguez-Trujillo

**Affiliations:** 1 Departamento de Patología Animal, Universidad de Las Palmas de Gran Canaria, Trasmontaña S/N, 35416 Arucas, Spain; oliver.rodriguez@ulpgc.es (O.R.); daniela.vazquez101@alu.ulpgc.es (D.V.); sara.delrosario@fpct.ulpgc.es (S.D.-R.); sergio.martin@ulpgc.es (S.M.); 2 Instituto Universitario de Investigaciones Biomédicas y Sanitarias, Universidad de Las Palmas de Gran Canaria, 35016 Las Palmas de Gran Canaria, Spain; yeray.brito@ulpgc.es; 3 Small Animal Surgery and Anaesthesia Service, Department of Veterinary Medicine, University of Teramo, 64100 Teramo, Italy; apaolini@unite.it; 4 Independent Researcher, 35016 Las Palmas de Gran Canaria, Spain; a.mateocebrian@gmail.com; 5 Unit of Reproduction, University Institute of Biomedical Research and Health, University of Las Palmas de Gran Canaria, Transmontaña S/N, 35413 Arucas, Spain; raquel.rodriguez@ulpgc.es

**Keywords:** sheep, ultrasound-guided peripheral nerve block, multimodal anaesthesia, bupivacaine, animal welfare

## Abstract

The demand for refined anaesthetic protocols in ovine experimental surgery has increased due to ethical considerations and the need to improve perioperative stability. This study evaluated the analgesic efficacy of ultrasound-guided combined sciatic and saphenous nerve blocks using two different local anaesthetics in Hair Canarian Sheep undergoing invasive orthopaedic hindlimb surgery. Fifteen clinically healthy sheep were randomly assigned to one of three groups: lidocaine (2%), bupivacaine (0.5%), or control (general anaesthesia alone). Intraoperative physiological parameters, including heart rate, respiratory rate, and arterial blood pressure, were recorded, and postoperative pain was assessed using a modified Melbourne Pain Scale. Sheep receiving locoregional anaesthesia showed significantly lower postoperative pain scores compared with control animals. Intraoperatively, a significant difference between groups was observed only for respiratory rate, with lower values in the bupivacaine group. The bupivacaine group exhibited lower and more stable respiratory rates, with a trend towards lower heart rates during surgery, as well as consistently lower pain scores during the early postoperative period. Lidocaine provided limited intraoperative and postoperative effects compared with the control group. Respiratory rate appeared to be more closely associated with pain scores than other physiological parameters. In conclusion, ultrasound-guided sciatic and saphenous nerve blocks were associated with improved perioperative analgesia in sheep undergoing orthopaedic surgery. The use of bupivacaine was associated with lower respiratory rates intraoperatively and reduced postoperative pain scores, suggesting a potential benefit in perioperative analgesia.

## Simple Summary

Sheep are widely used in experimental orthopaedic research, and ensuring effective pain management during and after surgery is essential for animal welfare and for the reliability of scientific results. General anaesthesia never provides adequate analgesia during invasive procedures and therefore requires the integration of additional analgesic strategies. This study evaluated whether combining general anaesthesia with ultrasound-guided nerve blocks of the sciatic and saphenous nerves could improve pain control in sheep undergoing hindlimb orthopaedic surgery. Two local anaesthetics, lidocaine and bupivacaine, were compared. Sheep receiving nerve blocks showed greater physiological stability during surgery and lower pain scores after surgery than animals receiving general anaesthesia alone. Among the two local anaesthetics, bupivacaine provided more consistent and longer-lasting pain relief than lidocaine. These results suggest that ultrasound-guided peripheral nerve blocks, particularly when performed with long-acting local anaesthetics, are an effective and practical approach to improve perioperative analgesia and animal welfare in sheep used for experimental orthopaedic procedures.

## 1. Introduction

The demand for refined anaesthetic protocols in veterinary practice and biomedical research has driven the advancement of multimodal strategies to improve perioperative care, particularly in species with unique physiological challenges such as ruminants. Small ruminants, especially sheep (Ovis aries), are widely used in translational research due to their manageable size, docile temperament, and anatomical similarities to humans in orthopaedic and implant studies [1,2,3]. Among these, the Hair Canarian Sheep breed has gained interest in experimental surgery due to its adaptability and availability in the Canary Islands.

Anaesthetic management in ruminants presents species-specific considerations that differ significantly from those in monogastric animals. Their high salivary output, susceptibility to regurgitation and aspiration, ruminal tympany, and ventilation–perfusion mismatch during recumbency make them particularly vulnerable during general anaesthesia. Ruminal tympany may severely compromise respiratory and cardiovascular function due to increased intra-abdominal pressure and diaphragmatic compression [4]. Moreover, the anatomical features of their airway complicate the process of endotracheal intubation, requiring careful technique and appropriate positioning to ensure airway protection and avoid aspiration pneumonia [5,6]. The physiological stress induced by these procedures can be minimized through the use of balanced anaesthetic protocols that combine general and regional techniques for improved patient stability [7,8]. Premedication with α2-adrenergic agonists such as xylazine is commonly incorporated into these protocols in ruminants because it improves sedation quality and contributes to balanced anaesthesia [9]. Although α2-adrenergic agonists such as xylazine are widely used in ruminants, their administration has been associated with hypoxaemia due to pulmonary effects. However, in the present study, oxygen supplementation and ventilatory support during anaesthesia may have mitigated these effects [9].

In line with current trends in veterinary anaesthesia, the integration of loco-regional anaesthetic techniques into multimodal protocols has shown considerable promise. Regional anaesthesia, particularly in orthopaedic procedures, enhances analgesia, reduces the requirement for inhalational agents, improves cardiovascular stability, and contributes to smoother recoveries [10,11]. Peripheral nerve blocks (PNBs), especially those targeting the sciatic and saphenous nerves, have emerged as effective techniques for hindlimb anaesthesia in companion animals and are increasingly being explored in ruminants [12]. These nerves innervate the majority of the pelvic limb distal to the mid-femur, making them suitable targets for blocking nociceptive input during orthopaedic interventions involving the tibia and stifle.

Ultrasound guidance has become the gold standard for the administration of PNBs, allowing for direct visualization of the needle and perineural structures, thereby increasing the precision and safety of the block [13,14]. Despite its advantages, the application of ultrasound-guided nerve blocks in sheep remains limited in the literature, with most available data extrapolated from studies in dogs, goats, and cattle [10,15,16]. Although nerve stimulation has traditionally been used to guide peripheral nerve blocks, ultrasound guidance has been shown to improve block accuracy by allowing direct visualization of neural structures, needle positioning, and local anaesthetic spread. Consequently, ultrasound-guided techniques are increasingly considered the preferred approach in both veterinary and human regional anaesthesia [16,17]. Furthermore, comparative information regarding the efficacy and duration of different local anaesthetic agents in sheep undergoing PNBs is scarce, especially under clinical surgical conditions.

The choice of local anaesthetic plays a critical role in the success of peripheral nerve blocks. Lidocaine, with its rapid onset and intermediate duration of action, is often preferred for short procedures, while bupivacaine offers a longer duration of analgesia but with a slower onset [18]. Understanding the clinical performance of these agents in ovine models is essential for both veterinary and translational applications. However, controlled comparative studies evaluating their intraoperative and postoperative analgesic efficacy in small ruminants are lacking.

In addition to providing adequate analgesia, objective and reliable pain assessment is fundamental for evaluating the efficacy of anaesthetic techniques. As prey species, sheep are known to exhibit stoic behaviour, often masking signs of discomfort or pain, particularly in the presence of humans [19]. Therefore, behavioural scoring systems, such as the adapted University of Melbourne Pain Scale, are essential tools for assessing pain in this species [20,21]. These multidimensional scales combine physiological, behavioural, and biological parameters to provide a more comprehensive evaluation of the animal’s pain status.

In this context, the present study aimed to evaluate the clinical efficacy of combined ultrasound-guided sciatic and saphenous nerve blocks in Hair Canarian Sheep undergoing invasive orthopaedic hindlimb surgery. Specifically, the study compared the intraoperative and early postoperative analgesic effects of two local anaesthetic agents—lidocaine and bupivacaine—based on cardiovascular parameters and multidimensional pain scoring. The findings aim to contribute to the refinement of perioperative analgesia in sheep used in experimental orthopaedic models. We hypothesized that ultrasound-guided peripheral nerve blocks, particularly with bupivacaine, would improve intraoperative stability and postoperative analgesia compared with general anaesthesia alone.

## 2. Materials and Methods

### 2.1. Animals

The study was conducted at the Veterinary Hospital of the University of Las Palmas de Gran Canaria (Spain), between March and November 2025. A total of fifteen clinically healthy Hair Canarian Sheep, comprising 5 males and 10 females, aged between 1 and 3 years and weighing between 40 and 50 kg, were included in the study. The animals were acquired from a local farm and acclimatized for at least one week prior to study onset. All sheep underwent a complete physical examination prior to enrolment and received routine antiparasitic treatment consisting of ivermectin (0.5 mL/25 kg SC; Vectimax^®^, Ceva Santé Animale, Libourne, France, 10 mg/mL), fenbendazole (5 mg/kg PO; Panacur^®^, Intervet International B.V., Boxmeer, The Netherlands, 100 mg/mL), and deltamethrin (10 mL topical; Butox^®^ Pour-On, Intervet International B.V., Boxmeer, The Netherlands, 7.5 mg/mL). Food was withheld for 12–24 h prior to anaesthesia to reduce the risk of regurgitation and aspiration, while water was available until 2 h before premedication. All procedures were performed in accordance with Directive 2010/63/EU and Spanish Royal Decree 53/2013 on the protection of animals used for scientific purposes. The study protocol was reviewed and approved by the Ethics Committee for Animal Experimentation of the Universidad de Las Palmas de Gran Canaria (CEEA-ULPGC) and authorized by the competent authority (approval code: OEBA_ULPGC 16/2024).

### 2.2. Experimental Design

This prospective, randomized, controlled, and blinded experimental study evaluated the analgesic efficacy of ultrasound-guided peripheral nerve blocks performed with two different local anaesthetics in sheep undergoing orthopaedic hindlimb surgery. Fifteen animals were randomly allocated to three groups (*n* = 5 each) using a computer-generated randomization list. A formal power analysis was not performed prior to the study because the work was designed as an exploratory experimental study using a limited number of animals. Sheep in the lidocaine group received ultrasound-guided sciatic and saphenous nerve blocks with 2% lidocaine at a total dose of 1 mg/kg, while animals in the bupivacaine group received the same blocks using 0.5% bupivacaine at the same dose. The dose of each local anaesthetic was calculated on a mg/kg basis for each animal. Consequently, the injected volume varied depending on the body weight of the animal and the concentration of the anaesthetic solution used. The control group received systemic analgesia without locoregional techniques.

All animals underwent a standardized unilateral tibial ostectomy with placement of a titanium scaffold. The same surgical team and anaesthetic protocol were used for all procedures, and nerve blocks were performed by the same experienced anaesthetist. All surgical procedures were performed on the left hindlimb, as the titanium scaffold used in the experimental model was specifically designed for implantation in that limb. Locoregional nerve blocks were performed by one anaesthetist, while intraoperative monitoring and data recording were carried out by a second anaesthetist who did not observe the block procedure and was unaware of which local anaesthetic had been administered. Postoperative pain assessment was performed by a third observer who was blinded to both the treatment allocation and the intraoperative findings.

Intraoperative physiological variables, including heart rate, respiratory rate, mean arterial pressure, oxygen saturation, and body temperature, were recorded. Postoperative pain was assessed at 2 and 4 h after extubation using a species-adapted version of the University of Melbourne Pain Scale. The requirement for rescue analgesia was recorded as a secondary outcome. All evaluations were performed by a single blinded observer. Physiological variables were recorded at predefined intraoperative timepoints corresponding to standardized surgical stages. Physiological variables were recorded every five minutes, and the values reported at each timepoint represent the mean of the measurements obtained during each surgical stage. T0 was defined as the period immediately after induction of anaesthesia and during performance of the ultrasound-guided nerve blocks; this pre-surgical phase lasted approximately 15 min. To minimize potential bias related to elapsed time, control animals were maintained under identical anaesthetic conditions for the same 15-min period before skin incision. T1 corresponded to skin incision and soft-tissue approach to the surgical site (approximately 10 min). T2 corresponded to the osteotomy stage (approximately 20 min). T3 corresponded to implant placement (plate fixation; approximately 15 min). T4 corresponded to layered wound closure (approximately 10 min). Animals with intraoperative complications or procedure times outside the predefined ranges were not included in the study to ensure comparable surgical duration across groups. The total duration of anaesthesia (from induction to discontinuation of inhalant anaesthesia) was approximately 90 min in all animals, and the surgical duration (from skin incision to completion of skin closure) was approximately 55 min. For postoperative assessments, time was referenced to discontinuation of inhalant anaesthesia (end of isoflurane administration), as this timepoint was standardized across animals and marked the start of recovery.

#### 2.2.1. Premedication and Induction of Anaesthesia

All sheep were premedicated intravenously with xylazine (0.05 mg/kg) and ketamine (0.4 mg/kg) and left undisturbed for 15 min. After adequate sedation, a 20 G intravenous catheter was aseptically placed in either the cephalic or saphenous vein. Anaesthesia was induced with intravenous propofol administered in incremental boluses up to a total dose of 1 mg/kg, titrated to effect until adequate conditions for endotracheal intubation were achieved. Endotracheal intubation was performed using a cuffed tube under direct laryngoscopic visualization. An orogastric tube was placed following intubation to reduce the risk of ruminal tympany and regurgitation. Baseline physiological parameters were recorded during the sedation period.

#### 2.2.2. Locoregional Anaesthesia

Ultrasound-guided peripheral nerve blocks of the sciatic and saphenous nerves were performed to provide locoregional anaesthesia of the pelvic limb. For the saphenous nerve block, animals were positioned in lateral recumbency with the surgical limb uppermost, and a linear ultrasound transducer was placed on the medial aspect of the mid-thigh to identify the femoral neurovascular bundle. The saphenous nerve was visualized as a hypoechoic structure caudal to the femoral artery, and a 22 G echogenic needle was advanced in-plane until positioned adjacent to the nerve. For the sciatic nerve block, the transducer was placed on the lateral aspect of the thigh, distal to the greater trochanter and ischiatic tuberosity, allowing identification of the sciatic nerve as a hypoechoic double-discoid structure. The needle was advanced in-plane towards the nerve under continuous ultrasound visualization. Animals in the lidocaine and bupivacaine groups received their respective local anaesthetics at a total dose of 1 mg/kg, divided between the two nerve blocks. No locoregional anaesthesia was performed in the control group. Block efficacy was indirectly assessed through intraoperative monitoring of nociceptive responses. Increases in heart rate or arterial blood pressure greater than 25% from baseline values were considered indicative of nociceptive stimulation and treated with rescue analgesia. All animals in the control group required a single intraoperative rescue administration, whereas no rescue analgesia was required in animals receiving locoregional nerve blocks. The effectiveness of the nerve blocks was further supported by the reduced postoperative pain scores observed in animals receiving locoregional anaesthesia.

#### 2.2.3. Maintenance of Anaesthesia

Anaesthesia was maintained with sevoflurane in oxygen delivered via a rebreathing system. After induction with 100% oxygen, FiO_2_ was reduced and maintained at approximately 50% using a mixture of oxygen and medical air. Mechanical ventilation using pressure support ventilation was applied throughout surgery, allowing spontaneous respiratory contribution, and adjusted according to capnography, respiratory mechanics, and oxygenation parameters. Physiological variables were monitored using a multiparameter veterinary monitor (Mindray ePM 12M Vet, Mindray Bio-Medical Electronics Co., Shenzhen, China), allowing continuous measurement of end-tidal carbon dioxide (EtCO_2_), inhalant anaesthetic gases, non-invasive arterial blood pressure (oscillometric method), heart rate, respiratory rate, oxygen saturation (SpO_2_), electrocardiography (ECG), and body temperature. Ventilatory parameters, including lung compliance, ventilatory pressures, and tidal volume, were obtained from the anaesthesia workstation (Mindray WATO EX-35 Vet, Mindray Bio-Medical Electronics Co., Shenzhen, China). Both the multiparameter monitor and the anaesthesia workstation were calibrated prior to each anaesthetic procedure according to the manufacturer’s recommendations. The anaesthesia workstation underwent a complete pre-use system check prior to each anaesthetic procedure, including verification of gas delivery, pressure, and flow measurements, in accordance with the manufacturer’s recommendations. And the multiparameter monitor underwent a pre-use functional check prior to each anaesthetic procedure, including verification of sensor performance, with internal automatic calibration systems operating according to the manufacturer’s specifications. Lactated Ringer’s solution was administered intravenously at 3 mL/kg/h. Heart rate, respiratory rate, arterial blood pressure, oxygen saturation, and rectal temperature were continuously monitored and recorded every five minutes. Arterial blood pressure was monitored using a non-invasive oscillometric method. The cuff was placed on the antebrachium and sized to approximately 40% of the circumference of the limb. An increase of ≥25% in heart rate or mean arterial pressure compared with baseline values was considered indicative of nociception and treated with intravenous butorphanol (0.05 mg/kg) as rescue analgesia. Butorphanol was selected as rescue analgesia due to its documented use as an opioid analgesic in large ruminant anaesthesia protocols and its favourable safety profile in veterinary anaesthesia [7].

#### 2.2.4. Recovery and Postoperative Care

Following surgery, all animals underwent postoperative radiographic evaluation to confirm implant positioning and were then transferred to individual recovery pens, where they were positioned in sternal recumbency as soon as possible. Extubation was performed after the return of swallowing reflexes. Postoperative treatment consisted of a single subcutaneous dose of meloxicam (0.2 mg/kg) and long-acting oxytetracycline (20 mg/kg), both administered immediately after extubation. Animals were continuously monitored during the postoperative recovery period in the university animal facility by trained personnel and under the supervision of the Animal Welfare Supervisor of the institutional Experimental Animal Facility, with real-time observation provided via a video surveillance system to detect abnormal behaviour or signs of discomfort.

### 2.3. Pain Assessment

Pain assessment was performed using an adapted version of the Melbourne Pain Scale incorporating the modifications proposed by Ahern [20]. This multidimensional scale is suitable for the evaluation of both conscious and semi-conscious animals and allows standardized quantification of nociceptive responses before and after surgical procedures. The assessment included physiological variables, together with biological indicators including pupillary dilation and salivation. Behavioural responses, namely reaction to palpation, locomotor activity, mental status, posture, and vocalization, were also considered. In its original formulation, the Melbourne Pain Scale yields a maximum score of 27 points, with pain intensity classified as absent, mild, moderate, or severe according to established thresholds. In the present study, the maximum achievable score was reduced to 22 points due to the exclusion of components not applicable to the experimental design. Pain assessment was performed only in conscious animals during the postoperative period. Rescue analgesia was planned to be administered if pain scores reached ≥10 points on the modified University of Melbourne Pain Scale during postoperative monitoring.

All pain evaluations were conducted by the same trained and blinded observer in order to minimize inter-observer variability and ensure consistency in scoring. Physiological variables included in the pain scale (heart rate, respiratory rate, arterial blood pressure, and rectal temperature) were obtained using the same multiparameter monitor described for intraoperative monitoring. Assessments were performed in a standardized sequence, beginning with non-invasive observation followed by direct interaction and palpation when required. Pain evaluations were conducted at 2 and 4 h after extubation.

#### Reference Physiological Parameters

Interpretation of physiological variables required consideration of normal reference values in sheep, as well as individual and environmental variability such as temperament, nutritional status, ambient temperature, or circadian influences [22,23]. Bradycardia was defined as HR < 55 bpm and tachycardia as HR > 140 bpm, considering the average baseline HR in adult sheep to be approximately 75 bpm [24]. Normal reference values for arterial blood pressure in sheep are as follows: SAP: 90–120 mmHg, DAP: 60–80 mmHg and MAP: 75–100 mmHg. Hypotension under general anaesthesia was defined as MAP < 65 mmHg, and hypertension as SAP > 120 mmHg or MAP > 100 mmHg [6,25].

### 2.4. Statistical Analysis

Statistical analyses were performed using StatPlus (AnalystSoft Inc., Walnut, CA, USA) for macOS. Normality of continuous variables was assessed by visual inspection of residuals and the Shapiro–Wilk test. Physiological variables recorded repeatedly over time (T0–T4) (heart rate [HR], respiratory rate [RR], systolic arterial pressure [SAP], diastolic arterial pressure [DAP] and mean arterial pressure [MAP]) were analyzed using a mixed (repeated-measures) ANOVA with treatment group (lidocaine, bupivacaine, control) as the between-subject factor, time as the within-subject factor, and the treatment × time interaction. Animals were treated as the subject factor to account for within-animal correlation across time. Data are presented as mean ± SD unless otherwise stated. Pain scores were treated as ordinal composite data and were analyzed using non-parametric methods. Pain scores are reported as median (min–max). Between-group comparisons at each postoperative time point (PAIN-1 and PAIN-2) were performed using the Kruskal–Wallis test. Within-group comparisons between PAIN-1 and PAIN-2 were performed using the Wilcoxon signed-rank test (two-tailed). A two-sided *p* value < 0.05 was considered statistically significant.

## 3. Results

The physiological parameters evaluated throughout the procedure demonstrated differences between the treatments (lidocaine, bupivacaine, and control) (Table 1).

All animals in the control group required a single intraoperative rescue analgesic administration due to increases in heart rate or arterial blood pressure exceeding 25% of baseline values, whereas no rescue analgesia was required in animals receiving locoregional nerve blocks.

Physiological variables recorded repeatedly over time (T0–T4) were evaluated using a mixed (repeated-measures) ANOVA with treatment group (lidocaine, bupivacaine, control) as the between-subject factor, time as the within-subject factor, and the treatment × time interaction. Respiratory rate (RR) showed a significant main effect of treatment group (*p* = 0.007), whereas the effects of time (*p* = 0.194) and the treatment × time interaction were not significant (*p* = 0.083). For heart rate (HR), there were no significant effects of treatment group (*p* = 0.248), time (*p* = 0.477), or their interaction (*p* = 0.626). Systolic arterial pressure (SAP) was significantly influenced by time (*p* < 0.001), with no effect of treatment group (*p* = 0.646) and no treatment × time interaction (*p* = 0.494). Similarly, diastolic arterial pressure (DAP) showed a significant effect of time (*p* < 0.001), with no effect of treatment group (*p* = 0.904) and no interaction (*p* = 0.086). Mean arterial pressure (MAP) also varied significantly over time (*p* < 0.001), while treatment group (*p* = 0.507) and the treatment × time interaction (*p* = 0.577) were not significant. Descriptive data for all physiological parameters are presented in Table 1.

The control group consistently presented the highest HR, with mean values ranging from 118 to 123 bpm across timepoints, while the bupivacaine group maintained lower and relatively stable HR values (92–93 bpm). In the lidocaine group, HR values were intermediate and showed slightly greater variability (100–114 bpm). Regarding respiratory rate, bupivacaine-treated animals exhibited the lowest RR throughout anaesthesia (17–20 bpm), with very narrow variation, with relatively low variability across timepoints. The control group showed the highest RR, especially during the initial timepoints (34–38 bpm). Lidocaine-treated animals had intermediate and slightly fluctuating RR values (31–34 bpm). In terms of mean arterial pressure (MAP), all groups showed relatively stable trends. However, the control group exhibited the highest MAP values (96–100 mmHg), while both bupivacaine and lidocaine groups showed lower pressures, ranging from 83 to 90 mmHg in bupivacaine and 74 to 89 mmHg in lidocaine.

Pain scores were analyzed using non-parametric methods and are presented as median (range) (Table 2). Pain scores differed significantly among treatment groups at both PAIN-1 (Kruskal–Wallis, *p* = 0.018) and PAIN-2 (Kruskal–Wallis, *p* = 0.015). Within each treatment group, pain scores did not differ between PAIN-1 and PAIN-2 (Wilcoxon signed-rank: lidocaine *p* = 0.590; bupivacaine *p* = 0.715; control *p* = 0.715).

## 4. Discussion

The present study demonstrates that the incorporation of ultrasound-guided combined sciatic and saphenous nerve blocks into a multimodal anaesthetic protocol may contribute to improved perioperative stability and postoperative analgesia in Hair Canarian Sheep undergoing invasive orthopaedic surgery. The present findings reinforce the growing evidence supporting the use of peripheral nerve blocks (PNBs) as refinement strategies in veterinary anaesthesia [26,27,28,29,30].

Ultrasound-guided sciatic and femoral/saphenous nerve blocks have been anatomically validated in sheep and shown to provide reliable limb desensitization [31]. Furthermore, ultrasound guidance improves block accuracy and reduces the risk of complications compared with landmark-based techniques [32]. The physiological stability observed in the present study aligns with previous reports demonstrating that regional anaesthesia reduces intraoperative stress responses and inhalant requirements in veterinary patients [33]. The benefits observed were related to analgesic efficacy and physiological stability rather than differences in anaesthetic duration, which was standardized across groups.

Pain assessment in sheep is inherently challenging due to their prey-species behaviour and tendency to mask clinical signs of discomfort. Recent literature highlights persistent gaps and opportunities in ovine pain management strategies [34]. The present findings suggest that respiratory rate may represent a more sensitive intraoperative marker of nociceptive activation than cardiovascular parameters alone.

These results are consistent with the pharmacological properties of the two agents. Lidocaine is characterized by rapid onset and intermediate duration, whereas bupivacaine provides prolonged sensory blockade due to its higher lipid solubility and protein binding [35]. One limitation of the present study is that both local anaesthetics were administered at an equal mg/kg dose despite known differences in their pharmacological potency. This approach may have favoured the longer-acting agent (bupivacaine) and should therefore be considered when interpreting the comparative analgesic effects observed. Comparative pharmacological discussions support the preferential use of longer-acting local anaesthetics when sustained postoperative analgesia is required [35].

Sheep are widely used in translational orthopaedic research due to their anatomical and biomechanical similarities to humans [36,37]. Their cortical bone structure, load-bearing characteristics, and bone remodelling dynamics make them valuable large-animal models for implant evaluation and regenerative strategies. However, perioperative nociception and postoperative severity may influence physiological responses capable of affecting experimental outcomes [38]. The implementation of ultrasound-guided sciatic and saphenous nerve blocks, therefore, represents both an ethical refinement aligned with the 3Rs framework and a methodological strategy to enhance experimental reproducibility.

In addition to improving perioperative analgesia, the use of regional anaesthesia techniques may contribute to the refinement of experimental orthopaedic models by minimizing nociceptive stress responses that could potentially influence physiological variables and experimental outcomes. The findings of the present study therefore support the incorporation of ultrasound-guided peripheral nerve blocks into anaesthetic protocols for sheep undergoing orthopaedic procedures, both as a strategy to enhance animal welfare and as a methodological approach to improve the reliability of translational research [15,37].

From a physiological perspective, peripheral nerve blocks may also offer advantages over neuraxial techniques. Epidural or spinal anaesthesia may induce sympathetic blockade and hypotension as a general physiological effect, although species-specific data in sheep are limited [33]. In contrast, peripheral nerve blocks act at the level of individual nerves and do not directly interfere with the sympathetic trunk to the same extent, generally resulting in effective sensory blockade with a more limited impact on systemic haemodynamics.

From a clinical standpoint, these characteristics make ultrasound-guided peripheral nerve blocks particularly attractive for orthopaedic procedures involving the pelvic limb, where targeted analgesia can be achieved while maintaining greater cardiovascular stability. The findings of the present study are consistent with this concept, as animals receiving sciatic and saphenous nerve blocks were associated with improved intraoperative physiological stability and lower postoperative pain scores compared with animals managed with general anaesthesia alone.

Furthermore, ultrasound guidance has been shown to improve block accuracy and reduce the risk of complications compared with landmark-based techniques [32]. The physiological stability observed in the present study is also consistent with previous reports demonstrating that regional anaesthesia may reduce intraoperative stress responses and inhalant anaesthetic requirements in veterinary patients [33].

Pain assessment in sheep remains inherently challenging due to their prey-species behaviour and their tendency to mask clinical signs of discomfort. Recent literature highlights persistent gaps and opportunities in ovine pain management strategies [34]. In the present study, respiratory rate appeared to correlate more closely with postoperative pain scores than other physiological variables, suggesting that it may represent a useful intraoperative indicator of nociceptive activation in this species.

The observed differences between treatment groups are consistent with the pharmacological properties of the two local anaesthetics evaluated. Lidocaine is characterized by a rapid onset and intermediate duration of action, whereas bupivacaine provides a longer-lasting sensory blockade due to its higher lipid solubility and protein binding [35]. Previous pharmacological studies have highlighted the advantages of longer-acting local anaesthetics when sustained postoperative analgesia is required [35].

Some limitations of the present study should be acknowledged. Both local anaesthetics were administered at an equal mg/kg dose despite known differences in their pharmacological potency. This approach may have favoured the longer-acting agent (bupivacaine) and should therefore be considered when interpreting the comparative analgesic effects observed. Future studies may benefit from dose-adjusted comparisons or pharmacodynamic evaluations designed to more precisely account for potency differences between local anaesthetics. Another limitation is the relatively small sample size, which reflects the ethical and logistical constraints inherent to experimental studies involving large-animal surgical models. Such limitations may reduce statistical power and the ability to detect more subtle differences between treatment groups. SpO_2_ provides only a limited assessment of oxygenation, particularly under oxygen supplementation, and arterial blood gas analysis would have provided a more precise evaluation. This represents another limitation of the present study.

In addition, postoperative pain assessment was limited to the early recovery period, and longer follow-up evaluations could provide further insight into the duration of analgesic effects and the potential development of delayed postoperative pain.

The implementation of ultrasound-guided sciatic and saphenous nerve blocks, therefore, represents both an ethical refinement aligned with the principles of the 3Rs and a methodological strategy to enhance experimental reproducibility. The integration of locoregional anaesthetic techniques into multimodal anaesthetic protocols may contribute not only to improved perioperative analgesia and animal welfare but also to the overall reliability of experimental orthopaedic research.

## 5. Conclusions

Ultrasound-guided combined sciatic and saphenous nerve blocks were associated with improved postoperative analgesia in Hair Canarian Sheep undergoing invasive orthopaedic hindlimb surgery. Intraoperatively, a significant effect between treatment groups was observed only for respiratory rate, with lower values in animals receiving bupivacaine.

The use of bupivacaine was associated with lower respiratory rates during anaesthesia and reduced postoperative pain scores, suggesting a potential benefit in perioperative analgesia. In contrast, lidocaine provided limited intraoperative and postoperative advantages compared with the control group.

These findings support the use of long-acting local anaesthetics as part of multimodal anaesthetic protocols in sheep, contributing to improved postoperative analgesia and refinement of experimental orthopaedic models.

## Figures and Tables

**Table 1:** Physiological parameters evaluated between different groups.

Physiological Parameters		LIDO	BUPI	CONTROL
HR (bpm)	T0	97 ± 24	102 ± 27	135 ± 11
	T1	114 ± 48	92 ± 25	122 ± 11
	T2	100 ± 43	92 ± 24	118 ± 9
	T3	102 ± 48	92 ± 26	122 ± 15
	T4	100 ± 50	90 ± 26	117 ± 20
RR (breaths/min)	T0	26 ± 10	20 ± 7	36 ± 2
	T1	34 ± 10	20 ± 6	38 ± 6
	T2	33 ± 10	17 ± 5	34 ± 6
	T3	31 ± 12	20 ± 5	34 ± 5
	T4	31 ± 12	19 ± 5	36 ± 6
SAP (mmHg)	T0	100 ± 19	106 ± 12	113 ± 23
	T1	104 ± 28	118 ± 11	124 ± 7
	T2	117 ± 30	123 ± 17	128 ± 4
	T3	122 ± 23	126 ± 21	124 ± 9
	T4	122 ± 18	126 ± 21	123 ± 8
DAP (mmHg)	T0	55 ± 25	54 ± 12	68 ± 23
	T1	60 ± 30	68 ± 16	74 ± 17
	T2	69 ± 33	69 ± 16	76 ± 20
	T3	75 ± 25	72 ± 18	71 ± 19
	T4	80 ± 20	73 ± 20	73 ± 17
MAP (mmHg)	T0	70 ± 21	72 ± 11	83 ± 22
	T1	74 ± 30	83 ± 15	96 ± 7
	T2	86 ± 30	89 ± 15	100 ± 6
	T3	89 ± 22	89 ± 19	97 ± 7
	T4	97 ± 19	91 ± 21	96 ± 9

Data are presented as mean ± SD (*n* = 5 per group). HR = heart rate; RR = respiratory rate; SAP/DAP/MAP = systolic/diastolic/mean arterial pressure. T0–T4 as defined in Methods.

**Table 2:** Pain scores (median [range]) at two postoperative time points (PAIN-1 and PAIN-2) in animals treated with lidocaine, bupivacaine, or control.

Group (*n* = 5)	PAIN-1 (Median [Range])	PAIN-2 (Median [Range])	*p* Value (Wilcoxon, PAIN-1 vs. PAIN-2)
Lidocaine	7 (5–8)	8 (5–9)	0.590
Bupivacaine	3 (3–7)	5 (3–5)	0.715
Control	8 (7–9)	8 (8–9)	0.715
Between-group *p*-value (Kruskal–Wallis)	0.018	0.015	

Within-group comparisons were performed using the Wilcoxon signed-rank test (two-tailed). Between-group comparisons were performed using the Kruskal–Wallis test.

## Data Availability

The original contributions presented in this study are included in the article. Further inquiries can be directed to the corresponding author.
